# Addressing catastrophic forgetting in payload parameter identification using incremental ensemble learning

**DOI:** 10.3389/frobt.2024.1470163

**Published:** 2024-11-04

**Authors:** Wael Taie, Khaled ElGeneidy, Ali Al-Yacoub, Ronglei Sun

**Affiliations:** ^1^ State Key Laboratory of Intelligent Manufacturing Equipment and Technology, School of Mechanical Science and Engineering, Huazhong University of Science and Technology, Wuhan, China; ^2^ School of Engineering, Coventry University, Cairo, Egypt; ^3^ Intelligent Automation Centre, Loughborough University, Loughborough, United Kingdom

**Keywords:** collaborative robots, payload dynamics identification, incremental learning, ensemble learning, catastrophic forgetting

## Abstract

Collaborative robots (cobots) are increasingly integrated into Industry 4.0 dynamic manufacturing environments that require frequent system reconfiguration due to changes in cobot paths and payloads. This necessitates fast methods for identifying payload inertial parameters to compensate the cobot controller and ensure precise and safe operation. Our prior work used Incremental Ensemble Model (IEM) to identify payload parameters, eliminating the need for an excitation path and thus removing the separate identification step. However, this approach suffers from catastrophic forgetting. This paper introduces a novel incremental ensemble learning method that addresses the problem of catastrophic forgetting by adding a new weak learner to the ensemble model for each new training bag. Moreover, it proposes a new classification model that assists the ensemble model in identifying which weak learner provides the most accurate estimation for new input data. The proposed method incrementally updates the identification model while the cobot navigates any task path, maintaining accuracy on old weak learner even after updating with new data. Validation performed on the Franka Emika cobot showcases the model’s superior accuracy and adaptability, effectively eliminating the problem of catastrophic forgetting.

## 1 Introduction

Machine learning has become a pivotal tool in addressing various tasks in the robotics field, offering accurate and reliable solutions to complex problems. Mimicking the human ability to identify payload inertial parameters rapidly or without the need for a separate identification process is one of the main goals in robotics, as addressed by machine learning techniques (Taie et al., 2023; Taie et al., 2024). This capability is crucial for cobots, as it allows them to adapt to dynamic manufacturing environments. The payload inertial parameters include payload mass, center of mass, and moment of inertia. Identifying these parameters is essential for achieving high-precision manipulation tasks, ensuring effective collision detection (Gaz and De Luca, 2017), and maintaining safety (Hamad et al., 2019), particularly in robots operated by model-based controllers.

Incremental ensemble machine learning that proposed in (Taie et al., 2024) provides the payload identification model with strong generalization abilities, enabling it to identify the payload without the excitation path required for batch learning models and traditional identification models. However, the incremental learning model suffers from the problem of forgetting old training data after updating with new data, a challenge known as catastrophic forgetting.

The key contributions of this work are:

• A novel incremental learning approach that addresses the catastrophic forgetting problem.
• A new classification model that assists the ensemble model in identifying which weak learner provides the most accurate estimation for new input data, thereby enhancing the overall accuracy of the ensemble model.
• Getting strong generalization ability for payload inertial parameter identification model by eliminating the need for specific excitation path while handling the problem of the catastrophic forgetting

The remainder of this work is structured as follows: Section 2 illustrates the literature review, Section 3 presents the proposed methodology, Section 4 explains payload inertial parameters identification using the proposed method, Section 5 illustrates the results, and Section 6 concludes the paper.

## 2 Literature review

Accurate identification of payload inertia parameters is critical across various robotic applications, significantly enhancing performance and safety. In medical robotics, for instance, tasks like Doppler sonography, minimally invasive surgery, and craniotomy demand high-precision torque control. (Sandoval and Laribi, 2022). developed a recursive least squares model to identify and compensate for tool inertia parameters, achieving an 80% improvement in torque controller precision. The identification process in this application follows an excitation trajectory lasting approximately 40 s. Similarly, (Winiarski et al., 2021), demonstrated that compensating payload parameters in a service robot that uses the impedance control enhances position error in the Z-axis by 70%, reducing the position error from 0.035 m to 0.01 m. In industrial applications, (Liu et al., 2024), highlighted the importance of accurate payload identification during slag removal processes, where tool parameter compensation decreased the standard deviation of measured force values, ensuring precise force reflection. The error in estimated tool weight of the proposed method is about 1.64%. (Gaz and De Luca, 2017). improved sensor less collision detection in collaborative robots by implementing online payload inertia parameter identification, achieving an estimation error of approximately 1% with an excitation trajectory lasting over 15 s. These applications underscore the significance need for advanced online identification techniques, such as ensemble machine learning.

Ensemble learning represents a robust machine learning approach that improves estimation accuracy by integrating the outputs of several models, or “learners,” leading to results that are more precise and resilient than those produced by any single model. The ensemble techniques discussed in (Ganaie et al., 2022; Dasari et al., 2023) encompass strategies such as bagging, boosting, and stacking, where models can function independently or in a sequential manner to enhance the ultimate prediction. According to data from the Scopus database, the number of academic papers focusing on ensemble learning in all disciplines has notably surged, increasing from 4,700 papers in 2010 to 88,350 papers in 2023 (Scopus, 2024). The utilization of ensemble machine learning is evident in diverse industries including healthcare for diagnosing diseases, finance for forecasting stock market trends, and cybersecurity for identifying potential risks (Dasari et al., 2023).

Ensemble learning techniques enhance the efficiency and performance of robotic arm applications through the integration of multiple algorithms for tasks such as movement control, calibration, and sensory perception. By employing a bioinspired model that utilizes ensemble learning, optimization of sensor and actuator duty cycles is achieved, resulting in decreased power consumption and improved control efficiency. Many algorithms are used to reach this aim such as Support Vector Machine, k-Nearest Neighbors, Naïve Bayes, Logistic Regression, and Multilayer Perceptron (Karlekar et al., 2023). Moreover, the application of ensemble learning contributes to enhancing the accuracy of industrial robot calibration, essential for precise positioning in intelligent manufacturing, by combining multiple calibration methods to achieve higher accuracy and robust generalization (Li et al., 2022). Additionally, ensemble learning plays a significant role in advancing sensory systems in robots, such as the electronic nose, which utilizes semiconductor and electrochemical sensors to accurately differentiate odors, even with limited training data, achieving an accuracy rate exceeding 90% (Li et al., 2021).

Our prior research (Taie et al., 2023) utilizes the batch ensemble learning method to tackle the challenge of identifying payload inertial parameters by avoiding noisy acceleration data, this approach enhances identification accuracy. It employs two different weak learners: a single-layer neural network (NN) and a basic decision tree (DT). The NN weak learner demonstrates higher accuracy when tested with real cobot data. Both weak learners are trained using the batch learning method with data measured by cobot joint sensors, including joint position, velocity, and torque measurements. During data collection, the cobot manipulates various payloads with different dynamic parameters.

The batch ensemble method proposed in (Taie et al., 2023) is similar to the conventional method described in (Kurdas et al., 2022; Kubus, Kröger, and Wahl, 2008; 2007; Salah et al., 2020; Sandoval and Laribi, 2022), which can only estimate the payload parameters while the cobot follows a specific excitation path. This ensures that the estimation process achieves the highest accuracy when the cobot adheres to the same excitation path. Deviations from this path excitation can lead to errors in the estimation results. The requirement for an excitation trajectory in both approaches causes delays in reconfiguring cobot settings for new tasks with different payloads, as the identification process must be conducted separately using the designated excitation path.

In small-batch production industries, it is common for cobot tasks to change frequently, with variations in both payload and task trajectory (Álvarez and Væhrens, 2022). Consequently, there is a need for a swift method to identify payload inertial parameters, which facilitates the rapid reconfiguration of cobot settings to match the unique requirements of each new payload. This agile methodology for task adaptation not only boosts the efficiency of cobot operations (Hu et al., 2020; Farsoni and Bonfè, 2022) but also streamlines production time by reducing delays linked to task transitions.

Our previous study (Taie et al., 2024) introduces a novel technique for determining the inertial parameters of payloads for collaborative robots (cobots) operating in dynamic manufacturing environments characterized by frequent reconfigurations. This technique eliminates the need for predefined excitation paths during the parameter identification process, enabling the cobot to identify parameters while following any arbitrary task path. The strategy utilizes an IEM that incorporates incremental neural networks as weak learners. This model adapts to new task paths while ensuring precise payload parameter estimations. Nevertheless, the model exhibits a tendency to forget previously learned information when updated with new task data, highlighting an area for further enhancement. This work serves as preliminary research for the current paper, where we address the problem of catastrophic forgetting. Catastrophic forgetting poses a substantial challenge in continual learning, where the performance of a model on previously acquired tasks diminishes as it learns new tasks (Ven et al., 2024).

Various strategies have been devised to tackle the catastrophic forgetting problem. Rehearsal-based approaches, which entail storing and replaying data from prior tasks, are effective but can lead to a task-recency bias. In this scenario, newer tasks overshadow older ones, leading to a decline in accuracy on earlier tasks (Wong, Koh, and Dobbie, 2023). Pseudorehearsal mechanisms generate synthetic data to mimic past experiences, alleviating data storage problems, yet they may not accurately replicate the original data distribution (Robins, 2022). Gradient-based optimization techniques demonstrate potential in retaining knowledge over long sequences of tasks, particularly when tasks reappear, but their effectiveness is constrained by the frequency of data reoccurrence (Lesort et al., 2022). Federated Class-Continual Learning (FCCL) strategies like TARGET employ exemplar-free distillation to transfer knowledge from previous to new tasks without storing actual data, thus preserving data privacy. However, they may struggle with non-independent and identically distributed data issues (Zhang et al., 2023). Transfer learning and knowledge distillation methods translate new data to reconstruct old data distributions and utilize old models to guide new ones, effectively mitigating forgetting but requiring meticulous implementation to prevent overfitting (Cheng et al., 2023).

Ensemble learning can effectively tackle the problem of catastrophic forgetting, as demonstrated in (Shi, 2023). The proposed ensemble model effectively prevents performance degradation in neural machine translation by combining multiple models and utilizing incremental learning techniques. Additionally, the Dynamic Scalable Self-Attention Ensemble (DSSAE) model introduced in (Ye and Bors, 2023), addresses catastrophic forgetting in Task-Free Continual Learning by dynamically adding Vision Transformer (ViT)-based experts based on sample novelty, eliminating the need for task-specific labels and ensuring optimal expert numbers. These methods collectively highlight the potential of ensemble learning to effectively mitigate catastrophic forgetting.

Despite the numerous techniques developed to address the issue of catastrophic forgetting, no method has yet been able to completely eliminate this problem, particularly in the context of payload parameter identification applications.

## 3 The proposed methodology

**FIGURE 1:**
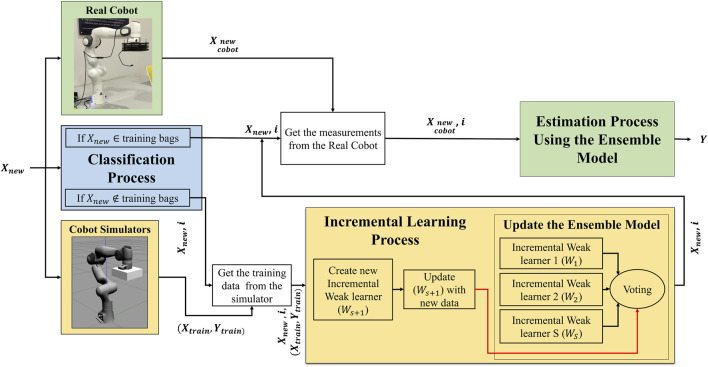
Proposed incremental ensemble method that handle the catastrophic forgetting problem.

The proposed methodology presented in Figure 1 aims at addressing the problem of catastrophic forgetting while performing incremental learning process. The methodology is divided into three main processes: classification, incremental learning and online estimation. The classification process checks if the new data point $X_{new}$ belongs to the data range of the previously trained model. The process moves to online estimation. If the point is outside the trained data range, the incremental learning process is triggered before estimation.

### 3.1 Classification process

The primary objective of the proposed classification process presented in Algorithm 1 is to segment the input data into clusters, or “bags,” based on predefined thresholds. Each bag can be visualized as a sphere, with the threshold representing its radius. These bags are formed incrementally and used to incrementally train specific weak learners, which are responsible for estimation process for any new points that fall within their respective spaces. When the new data point $X_{new}$ is given to the algorithm, it is classified online to determine whether it belongs to or is distant from the space of the existing bags. If $X_{new}$ does not fit within any current bag, it will be used to form a new bag.

Algorithm 1 Data classification and online bags formation.

1. **Inputs:**New data point $\left(X_{new}\right)$ ,predetermined thresholds $\left(\sigma \right)$

2. **Outputs:**list of bags (bags), vector of mean value of each bag ( V ), nearest bag index ( $\left.i\right)$

3. Initialize an empty bag
4. Initialize an empty V

5. for each new data point $X_{new}$ do
6. $X_{new}$ belongs to bag space = False
7.   if V is not empty then
8.    for each mean value ${\bar{V}}_{s}$ in V do
9.     Calculate the Euclidian distance $d_{s}$

10.     Add $d_{s}$ to Euclidian distance vector d

11.     if $d_{s}$ < σ then
12.      Add $X_{new}$ to ${bag}_{s}$

13.      Calculate the mean value of ${bag}_{s}$ as ${\bar{V}}_{s}$

14.      Update the value of ${\bar{V}}_{s}$ in V

15. $X_{new}$ belongs to bag space = True
16. i = index (min ( d ))
17.     break
18.    end if
19.   end for
20.  end if
21. if $X_{new}$ belongs to bag space = False then
22.   Create a new bag
23.   Add $X_{new}$ to this new bag
24.   Add the new bag to bags
25.   Calculate the mean value of the new bag as ${\bar{V}}_{new}$

26.   Add ${\bar{V}}_{new}$ to V

27. $X_{new}$ belongs to bag space = True
28. i = index ( V ( ${\bar{V}}_{new}$ ))
29.  end if
30. end for

### 3.2 Incremental learning process

The objective of the proposed incremental ensemble learning process is to update the model with new data $X_{new}$ without encountering the catastrophic forgetting problem. The proposed method shown in Algorithm 2 consists of two steps. The first step involves incremental learning by generating a new weak learner for the new bag by copying the nearest bag’s weak learner. Once the new bag and weak learner are established, the process proceeds to the second step, in which incremental learning is performed while the new bag is formed. Incremental learning during the formation of a new bag is achieved by updating the weights of the weak learner based on the new data. The weak learners in the proposed IEM are single-layer neural networks that are incrementally trained with new data by the Stochastic Gradient Descent (SGD) optimization algorithm. SGD is selected for its fast computation speed (Zhou et al., 2021).

### 3.3 Estimation process

The proposed ensemble model comprises several weak learners, the most accurate output is the output of the weak learner trained with $X_{new}$ training data or the output of the weak learner that $X_{new}$ falls into its bag space. So, the output vector Y of the ensemble model is determined as follows in Equation 1:

$$Y=\sum_{s=1}^{I}n_{s}{\hat{Y}}_{s}$$ (1)

where $Y_{s}$ is the inertial parameters estimated from weak learner s . The selection factor of the weak learner s , denoted as $n_{s}$ is defined as follows in Equation 2:

$$n_{s}=\left\{\begin{array}{l} 1,\;if\;index\left(nearest\;bag\right)=s\\ 0\;,\;\mathrm{else} \end{array}\right.$$ (2)

## 4 Payload inertial parameters identification using the proposed method

In this section, the proposed method is adapted to solve the problem of payload inertial parameters identification. In this framework, real cobot measurements are employed for estimation, while cobot simulators with training payloads are used for collecting data for incremental learning. Both real cobots and simulators work concurrently to perform the new task path.

**FIGURE 2:**
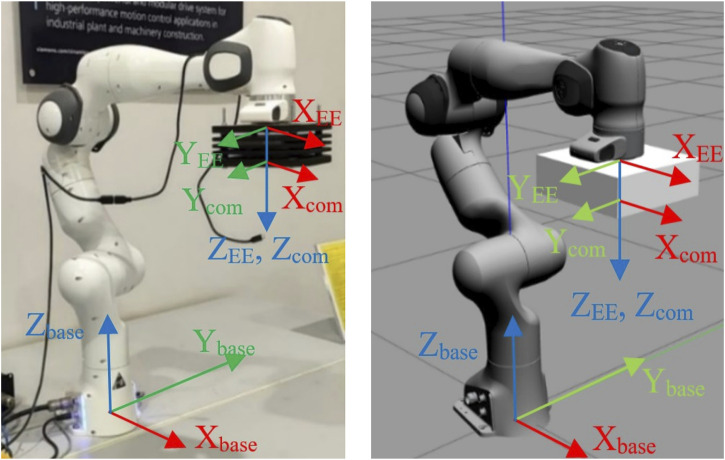
Experimental setup.

The Franka Emika Cobot, which features seven revolute joints and a maximum payload capacity of 3 kg, serves as the test platform for this methodology. The setup for both the simulation and the real cobot is identical, as depicted in Figure 2. The payload is securely mounted on the cobot’s end effector, ensuring that the base frame, end effector frame, and payload center of mass frame are consistent between the simulation and the real cobot. This uniformity in setup allows the simulation environment to accurately collect training data for the payload currently attached to the real cobot’s end effector, with both the simulator and the real cobot sharing the same configuration and moving in tandem.

**FIGURE 3:**
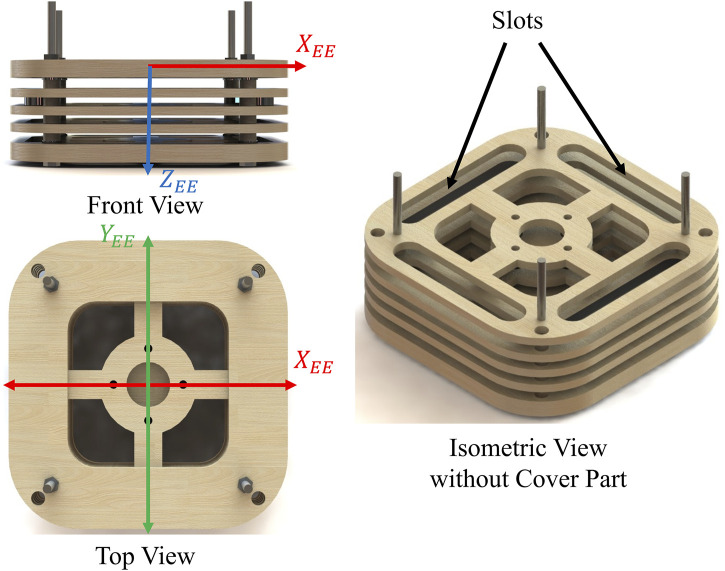
Adjustable payload design.

The payload used in the training process and testing experiments is sourced from (Taie et al., 2023). It is designed to permit independent adjustments of mass and center of mass components. The setup, shown in Figure 3, features a container with four slots for three metal weights each. Each weight is 0.275 kg, and the container weighs 0.790 kg. Changing weight placement affects $r_{x}$ ., $r_{y}$ , or both, while position shifts within one slot adjust $r_{z}$ . The overall payload mass can vary by altering the number of weights used. This design accommodates various payload configurations for simulators. In the training data collected from the simulator, the payload mass starts with the empty container’s weight and increases by adding weights incrementally, up to five. The total mass with five weights reaches 2.163 kg, staying within the maximum payload limit. This method ensures operational safety and prevent reflex errors in the cobot controller during variable trajectories. A total of 77 unique payload are used to ensure diversity of the training data.

### 4.1 Path classification

The input of our algorithm is new path points $q_{new}$ in joint space, denoted by Equation 3, which is used with the cobot’s forward kinematic model to calculate the end effector’s position in Cartesian space, as illustrated in Equation 4.

$$q_{new}=\left[q_{0_{new}},q_{1_{new}},q_{2_{new}},q_{3_{new}},q_{4_{new}},q_{5_{new}},q_{6_{new}}\right]$$ (3)

$$R_{new}=FK\;\left(q_{new}\right)=\left(X_{new},Y_{new},Z_{new}\right)$$ (4)

In addition, the mean vectors of existing bags in joint space and in cartesian space are stored as presented in Equations 5, 6 where s refers to the training bag index.

$${\bar{q}}_{s}=\left[{\bar{q}}_{0_{s}},{\bar{q}}_{1_{s}},{\bar{q}}_{2_{s}},{\bar{q}}_{3_{s}},{\bar{q}}_{4_{s}},{\bar{q}}_{5_{s}},{\bar{q}}_{6_{s}}\right]$$ (5)

$${\bar{R}}_{s}=\left({\bar{X}}_{s},{\bar{Y}}_{s},{\bar{Z}}_{s}\right)$$ (6)

The Euclidean distance vectors in joint space ( $d_{s}$ ) and in cartesian space $\left(K_{s}\right)$ is calculated between the new point data ( $q_{new}$ and $R_{new}$ ) and the mean vectors ( ${\bar{q}}_{s}$ and ${\bar{R}}_{s}$ ) for every bag as shown in Equations 7, 8.

$$d_{s}\left(q_{new},{\bar{q}}_{i_{s}}\right)=\sqrt{\sum_{i=1}^{j}{\left(q_{i_{new}}-{\bar{q}}_{i_{s}}\right)}^{2}}$$ (7)

$$K_{s}\left(R_{new},{\bar{R}}_{s}\right)=\sqrt{{\left(X_{new}-{\bar{X}}_{s}\right)}^{2}+{\left(Y_{new}-{\bar{Y}}_{s}\right)}^{2}+{\left(Z_{new}-{\bar{Z}}_{s}\right)}^{2}}$$ (8)

The predetermined threshold in the joint space is $\left(\delta \right)$ and in Cartesian space is ( ρ ). If ( $d_{s}$ < δ ) and ( $K_{s}$ < ρ ), it means the $q_{new}$ belongs to the space of bag (s) at that time the measured data of point $q_{new}$ in the real cobot can be used directly with the estimation process. If this condition is not true, the $q_{new}$ and $R_{new}$ will be used to form a new bag incrementally based on the predetermined thresholds till the end of this task path.

**FIGURE 4:**
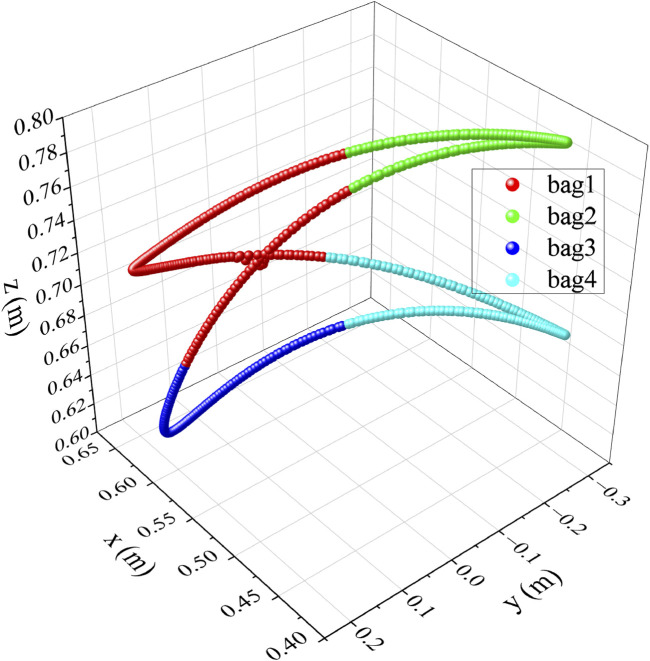
Classification process result for the cobot path in cartesian space.

The process of creating the initial training bags is applied by using the excitation path sourced from (Taie et al., 2023). The classification process results are presented in Figure 4. The path data is automatically divided into 4 bags based on δ value that equal 30° and ρ value that equal 0.2 m. The new bags can be added based on the values of the thresholds. Each bag has its own mean value that accurately representing that particular segment of the path. Only the mean value of each bag will be stored to be used in classifying the new coming points.

### 4.2 The payload parameters identification process

The identification process is initiated immediately after the classification process, but only if the new path point falls within the space of an existing training bag. The proposed IEM is employed to estimate the payload inertial parameters online, utilizing real cobot measurements. The output vector of the estimation model is defined as follows in Equation 9:

$$\phi =\left[m,mr_{x},mr_{y},mr_{z},I_{xx},I_{xy},I_{xz},I_{yy},I_{yz},I_{zz}\right]$$ (9)

The input vector (X) of the proposed estimation model consists of joint angles $\left(q_{new}\right)$ , joint velocities $\left({\dot{q}}_{new}\right)$ and joint torques $\left(\tau_{new}\right)$ as detailed in  Equations 10–12.

$$q_{new}=\left[q_{0_{new}},q_{1_{new}},q_{2_{new}},q_{3_{new}},q_{4_{new}},q_{5_{new}},q_{6_{new}}\right]$$ (10)

$${\dot{q}}_{new}=\left[{\dot{q}}_{0_{new}},{\dot{q}}_{1_{new}},{\dot{q}}_{2_{new}},{\dot{q}}_{3_{new}},{\dot{q}}_{4_{new}},{\dot{q}}_{5_{new}},{\dot{q}}_{6_{new}}\right]$$ (11)

$$\tau_{new}=\left[{\tau_{0}}_{new},{\tau_{1}}_{new},{\tau_{2}}_{new},{\tau_{3}}_{new},{\tau_{4}}_{new},{\tau_{5}}_{new},{\tau_{6}}_{new}\right]$$ (12)

The training process described by Algorithm 2 is repeated for every task path point ( $q_{new}$ ) that falls outside the space covered by the old training dataset. Once the training is complete, the input data for the path point ( $q_{new}$ ) is measured from the real cobot joints. This data is then used in the estimation process with the updated model. The cobot simulators operate simultaneously with the real cobot to provide training data for the point $\left(q_{new}\right)$ . The training sample at this point consists of an array of input data (X) and output data ( ϕ ) from 77 training payloads, sourced from (Taie et al., 2023). The optimization of the weak learner parameters is performed incrementally by iterating over the 77 training points.

Algorithm 2 The Proposed incremental learning algorithm.

1.  **Inputs:**New data point $X_{new},$ the new training Sample $\left(X,Y\right)$ , nearest bag index ( i ), weak learners ( W )
2.  **Output:**Estimated output $\hat{Y}$

3.  Initialize the nearest weak learner $W_{i}=W\left(i\right)$

4.  Create a new weak learner $W_{new}=copy\left(W_{i}\right)$

5. $W_{new}=\text{Incrementallearning}\left(W_{new},\left(X,Y\right)\right)$

6. $\hat{Y}=W_{new}.predict\left(X_{test}\right)$

**Function:** Incrementallearning

7.  **Inputs:**weak learner $W_{new}$ , training sample $\left(X,Y\right)$

8.  **Output:**Updated weak learner $W_{updated}$

9.  Initialize learning rate α

10.  Initialize activation function $ReLU\left(\right)$

11. $W_{new}\;\to \;\hat{Y}=ReLU\left(aX+b\right)$

12. $L=\frac{1}{N}\sum_{k=1}^{N}{\left(Y_{k}-{\hat{Y}}_{k}\right)}^{2}$

13. $\nabla_{a}L=\frac{\partial L}{\partial a},\nabla_{b}L=\frac{\partial L}{\partial b}$

14. $a_{new}=a_{old}-\alpha \nabla_{a}L$

15. $b_{new}=b_{old}-\alpha \nabla_{b}L$

16. $W_{updated}\;\to \;\hat{Y}=ReLU\left(a_{new}X+b_{new}\right)$

17.  **End Function**

## 5 Results

The results presented in this section evaluate the performance of the proposed Incremental Ensemble Model (IEM) in identifying payload inertial parameters, The real testing payload parameters is shown in Table 1. The testing payload parameters are entirely new. These unseen parameters are crucial in assessing the model’s ability to generalize to previously unseen data. The experiments were designed to test the model’s accuracy and its ability to mitigate the problem of catastrophic forgetting, comparing it against the Batch Ensemble Model (BEM) and an older Incremental Ensemble Model. The key metrics used to assess performance include the Mean Absolute Error (MAE) for parameters such as mass, center of mass, and moment of inertia The machine used to perform the experiments operates on the Ubuntu operating system, boasting 8-core CPU running at 2.30 GHz, complemented by 16 GB memory capacity.

**TABLE 1:** The inertial parameters of the real testing payload.

Parameters	m	$mr_{x}$	$mr_{y}$	$mr_{z}$
Unit	(kg)	(kg.m)		
values	1.34011	−0.01608	0.01608	0.03654

Parameters	$I_{xx}$	$I_{xy}$	$I_{xz}$	$I_{yy}$	$I_{yz}$	$I_{zz}$
Unit	(kg.m^2^)					
values	0.00742	0	−0.00108	0.00742	0.00108	0.00982

### 5.1 Identification results comparison between proposed IEM and prior models using the excitation path data

The aim of this section is to ensure that the accuracy of the proposed IEM matches that of the batch ensemble learning model proposed in (Taie et al., 2023) and the previous incremental model proposed in (Taie et al., 2024) in estimating payload inertial parameters. The training and testing of the three models were conducted using the same excitation path data shown in Figure 3. The mean absolute error (MAE) for the three models are compared in Table 2. Notably, all models demonstrated a comparable level of accuracy across most inertial parameters. However, the proposed IEM significantly outperforms the batch ensemble model (BEM) and the old IEM in predicting some of inertia tensors parameters such as $I_{xx}$ $,I_{yz}$ and $I_{zz}$ .

**TABLE 2:** Comparison of MAE of Estimation of Payload Inertial Parameters Between BEM, Old IEM and proposed IEM, all trained and tested by the excitation path.

Parameters		m	$mr_{x}$	$mr_{y}$	$mr_{z}$
Unit		(kg)	(kg.m)		
MAE	BEM	0.00649	0.005377	0.004011	0.00301
	Old IEM	0.00655	0.006354	0.004695	0.007396
	Proposed IEM	0.00657	0.007127	0.00654	0.003422

Parameters		$I_{xx}$	$I_{xy}$	$I_{xz}$	$I_{yy}$	$I_{yz}$	$I_{zz}$
Unit		(kg.m^2^)					
MAE	BEM	0.019836	0.006947	0.003738	0.00649	0.0047	0.00369
	Old IEM	0.000662	0.000432	0.000637	0.000968	0.001538	0.000568
	Proposed IEM	0.000382	0.00102	0.001033	0.00101	0.000364	0.000267

### 5.2 Identification results comparison between proposed IEM and prior models using the novel path data

The objective of this section is to compare the MAE of the proposed IEM, old incremental model and the batch learning model when the cobot follows a novel linear path. The start and final positions of this path are shown in Table 3. Unlike the sinusoidal excitation path used during training, this task path is planned to move all cobot joint angles linearly to reach the final position. The estimation results in Table 4 show that the BEM totally fails to identify any of the payload parameters with a large value of the mean absolute error through the path. The proposed and the old incremental ensemble methods succeeded in adapting the model with the new path data and giving accurate estimations for the payload parameters. The MAE of the proposed method estimations for mass, center of mass and the moment of inertia parameters are 0.007 (kg), 0.008 (kg.m) and 0.0007 (kg.m^2^) respectively.

**TABLE 3:** The new task path start position and final position are represented in joint space.

Joint angles	*q*_*0*_	*q*_*1*_	*q*_*2*_	*q*_*3*_	*q*_*4*_	*q*_*5*_	*q*_*6*_
Start position (degree)	0	−45	0	−135	0	90	45
Final position (degree)	−12	52	−14	−96	19	146	8

**TABLE 4:** Comparison of MAE of estimation of payload inertial parameters between BEM, Old IEM and proposed IEM while the cobot follows novel task path.

Parameters		m	$mr_{x}$	$mr_{y}$	$mr_{z}$
Unit		(kg)	(kg.m)		
MAE	BEM	13.2357	5.6501	1.7456	2.8403
	Old IEM	0.010036	0.005612	0.006282	0.008791
	Proposed IEM	0.007293	0.0084387	0.009467	0.006906

Parameters		$I_{xx}$	$I_{xy}$	$I_{xz}$	$I_{yy}$	$I_{yz}$	$I_{zz}$
Unit		(kg.m^2^)					
MAE	BEM	0.1606	0.0003	0.0053	0.1705	0.0113	0.2528
	Old IEM	0.000662	0.000432	0.000637	0.000968	0.001538	0.000568
	Proposed IEM	0.00056	0.000445	0.001164	0.000953	0.000674	0.00043

### 5.3 Evaluating the forgetting problem in the proposed IEM and prior models

**FIGURE 5:**
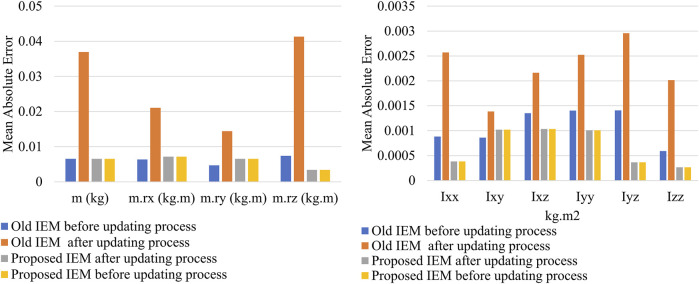
Comparison between MAE of the IEMs before and after the update process, the test is performed using the old excitation path data.

In this section, we evaluate the proposed algorithm’s ability to address the problem of catastrophic forgetting. A comparison is made between the old IEM and the proposed IEM. Both models are tested twice using the old excitation path data: once before updating with new path data and once after. The results in Figure 5 show that the MAE of the mass, center of mass, and moment of inertia parameters for the old IEM increases to 0.037 (kg), 0.025 (kg.m), and 0.0022 (kg.m2), respectively, after the update. In contrast, the proposed IEM maintains the same level of accuracy in estimating all parameters both before and after the update.

A limitation of the proposed method is its tendency to increase the size of the ensemble model. Specifically, the number of bags and weak learners will continue to grow until the entire cobot working space is covered. Experimental results indicate that the time (T) required to determine whether a new point belongs to a specific bag is approximately 0.001 s. Consequently, as the number of bags increases, the total classification time increases linearly without affecting the accuracy of the model. However, storing only the center of each bag will reduce the amount of data required to be stored. Also, the threshold value can reduce the number of formed bags.

### 5.4 Identification time for the proposed IEM

In this section, we evaluate the identification time. According to the classification process, if a new point belongs to one of the existing bag spaces, it is sent directly to the identification process, which is performed instantaneously. This instantaneous response is one of the advantages of ensemble learning identification, as explained by (Taie et al., 2023). Our experiment verifies this, with the identification time for each single new point being just 0.003 s.

**FIGURE 6:**
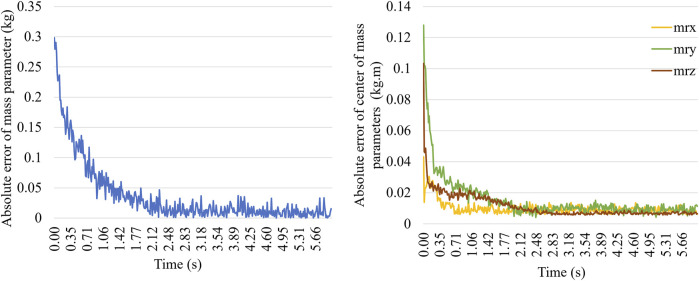
Identification time of the new task path.

However, if the new point does not belong to an existing bag, a new bag begins to form, and a new weak learner is updated with the new data. In this case, the process of online estimation is performed for each point, with each new point taking about 0.012 s to identify the payload parameters. Figure 6 shows the absolute error of the online estimation of the payload mass and center of mass *versus* time as the cobot follows the new task path. The total task path time is 6 s. The convergence time for the estimation of the mass and center of mass parameters is approximately 2 s, while the convergence time for the moment of inertia parameters is around 1 s.

## 6 Conclusion

The proposed IEM effectively adapts to new task paths, maintaining a low MAE for parameters such as mass, center of mass, and moment of inertia, even with the introduction of new task data. Furthermore, the proposed IEM mitigates the catastrophic forgetting problem more effectively than old IEM by incorporating new weak learners updated with the new task data, rather than merely adjusting the weights of the original weak learners. Old IEM experienced increased MAEs for all parameters after updating, whereas the proposed method maintained consistent accuracy levels.

Additionally, while the BEM struggled to accurately identify payload parameters along new task paths, resulting in high MAE values, the incremental models achieved significantly lower MAEs, ensuring better performance and reliability.

However, a notable limitation of the proposed method is the potential for continuous growth of the ensemble model, as new weak learners are added with each new task path until the entire cobot working space is covered, leading to increased computational and storage demands over time. Despite this limitation, the proposed IEM offers substantial improvements in adapting to new tasks and maintaining accuracy without experiencing catastrophic forgetting. Future work may focus on optimizing the model’s size to balance accuracy and resource efficiency.

## Data Availability

The original contributions presented in the study are included in the article/supplementary material, further inquiries can be directed to the corresponding author.
